# An energy metabolism-based eight-gene signature correlates with the clinical outcome of esophagus carcinoma

**DOI:** 10.1186/s12885-021-08030-0

**Published:** 2021-04-01

**Authors:** Weifeng Zheng, Chaoying Chen, Jianghao Yu, Chengfeng Jin, Tiemei Han

**Affiliations:** 1 The department of Gastroenterology, the Forth Affiliated Hospital Zhejiang University School of Medicine, No. N1, Shangcheng Avenue, Yiwu City, 322000 Zhejiang Province China; 2 The department of Cardio-Thoracic Surgery, the Forth Affiliated Hospital Zhejiang University School of Medicine, Yiwu, China

**Keywords:** Esophagus cancer, Energy metabolism, Signature, Risk score, Prognosis

## Abstract

**Background:**

The essence of energy metabolism has spread to the field of esophageal cancer (ESC) cells. Herein, we tried to develop a prognostic prediction model for patients with ESC based on the expression profiles of energy metabolism associated genes.

**Materials and methods:**

The overall survival (OS) predictive gene signature was developed, internally and externally validated based on ESC datasets including The Cancer Genome Atlas (*TCGA*), GSE54993 and GSE19417 datasets. Hub genes were identified in each energy metabolism related molecular subtypes by weighted gene correlation network analysis, and then enrolled for determination of prognostic genes. Univariate, LASSO and multivariate Cox regression analysis were applied to assess prognostic genes and build the prognostic gene signature. Kaplan-Meier curve, time-dependent receiver operating characteristic (ROC) curve, nomogram, decision curve analysis (DCA), and restricted mean survival time (EMST) were used to assess the performance of the gene signature.

**Results:**

A novel energy metabolism based eight-gene signature (including UBE2Z, AMTN, AK1, CDCA4, TLE1, FXN, ZBTB6 and APLN) was established, which could dichotomize patients with significantly different OS in ESC. The eight-gene signature demonstrated independent prognostication potential in patient with ESC. The prognostic nomogram constructed based on the gene signature showed excellent predictive performance, whose robustness and clinical usability were higher than three previous reported prognostic gene signatures.

**Conclusions:**

Our study established a novel energy metabolism based eight-gene signature and nomogram to predict the OS of ESC, which may help in precise clinical management.

**Supplementary Information:**

The online version contains supplementary material available at 10.1186/s12885-021-08030-0.

## Background

Esophageal cancer (ESC) is the seventh most common cancer globally [1]. The prognosis of advanced ESC is still not satisfactory and treatment options are limited [2, 3]. Most first-line chemotherapy for advanced esophageal cancer adopt platinum combined or fluorouracil-based regimens, with an effective rate of 40–60% [1]. However, the median overall survival (OS) time of patients after failure of first-line treatment is only 5–10 months, and there is no standard effective treatment regimen for second-line treatment [1]. Despite comprehensive genomic characterization [4], available target therapies for ESC are still lagging behind [1], therefore, in order to prognosticate patient’s clinical outcome, increasing interest was focused on the molecular characterization of ESC.

The interaction of tumor cells and energy metabolism is supposed to play an important role in ESC progression [5, 6]. Warburg effect and active glutaminolysis are oncogene-driven mechanism that alters the metabolism of cancer cells, and they are presumed to be the hallmarks of tumorigenesis [7]. Cancer-associated metabolic remodeling is considered as the direct response to cell growth and survival signals [8]. Thus, the detection of cellular metabolites can provide promising diagnostic and prognostic biomarkers. For example, several metabolites have been identified as prognosis predictive features for patients with ESC [9]. Although cumulative evidences confirm that multiple energy metabolism related genes participate in the malignant behaviors of ESC cells, such as proliferation, metastasis, angiogenesis, drug sensitivity, etc., and some of them are identified as prognostic predictor in ESC [10–13]. The potential molecular typing and prognostic value of the expression characteristics of energy metabolism-related genes (EMRGs) in ESC has not be comprehensively analyzed yet.

The present study aimed at the detection of new molecular subtype and prognostic signature in patients with ESC. The Cancer Genome Atlas (*TCGA*) ESC dataset was used to analyzed the expression profile of energy metabolism related genes. A total of 472 mRNAs related to energy metabolism were analyzed in this study and an eight-gene signature was established that can effectively predict clinical outcome of patient with ESC, which were independent validated in the GSE54993 and GSE19417 dataset.

## Materials and methods

### Datasets and gene sets

A total of 78 ESC samples with clinical information and RNA-seq data from *TCGA* database was collected. *TCGA* database (http://portal.gdc.cancer.gov/) was collected with the accession number TCGA-ESCA, the TCGA ESC dataset was pre-processed with the criteria as follows: 1) excluded samples absent of clinical data and overall survival (OS) < 30 days; 2) excluded data of normal esophagus tissue sample; 3) excluded genes with Fragments Per Kilobase of exon model per Million mapped fragments (FPKM) = 0 in 50% of cases; and 4) included the expression profile of genes associated with energy metabolism. Besides, we found two datasets from the NCBI Gene Expression Omnibus (GEO) database (http://www.ncbi.nlm.nih.gov/geo/), the accession number were GSE54993 and GSE19417. GSE54993 dataset, which contains CGH data of ESC [4], and GSE19417 dataset, which contains gene expression profiles of ESC [14]. GSE54993 and GSE19417 datasets were pre-processed with the criteria as follows: 1) excluded normal tissue sample data; 2) transformed gene probes to the human gene SYMBOL, removed those probes matched to multiple genes, if several probes matched to one gene, the median was selected as the expression profile of this gene; 3) normalized microarray data by using Robust Multi-Array Average method [15]. The clinicopathological features of patients from these three datasets after preprocessing are showed in Table 1. For *TCGA* ESC dataset, 75% of them was randomly divided into training cohort (*n* = 58), and the entire dataset was selected as internal validation cohort. The GSE54993 (*n* = 70) and GSE19417 (*n* = 70) datasets were applied as external validation cohorts. This study was approved by the Institutional Review Boards (IRB) of the Fourth Hospital affiliated to Zhejiang University.

**Table 1:** Clinical and pathologic characteristics of patients in the pre-processed *TCGA* and GEO datasets

Characteristic		*TCGA* training dataset (*n* = 58)	*TCGA* entire dataset (*n* = 78)	*p* value	GSE54993 dataset (*n* = 70)	GSE19417 dataset (*n* = 70)
Age (years)	≤55	26	35	1	28	NA
	> 55	32	43		42	NA
Survival Status	Living	42	54	0.831	34	13
	Dead	16	24		36	57
Gender	female	7	12	0.762	13	24
	male	51	66		57	46
Smoking history	NO	18	27	0.841	26	NA
	YES	37	48		44	NA
pT stage	T1	4	7	0.874	4	NA
	T2	20	28		2	NA
	T3/T4	34	43		64	NA
pN stage	N0	34	41	0.597	32	NA
	N1/NX	24	37		38	NA
pM stage	M0	49	67	0.866	70	NA
	M1/MX	9	10		0	NA
pTNM stage	Stage I	5	6	0.946	5	NA
	Stage II	35	46		29	NA
	Stage III	14	23		36	NA
	Stage IV	3	4		0	NA

*NA* not available

### Identification of molecular subtypes based on EMRGs

Coherently expressed signatures of 11 human metabolism-related pathways (Table 2), all download from the Reactome pathway database (https://reactome.org/) [16], were derived by aggregating MSigDB version 7.0 gene sets. A total of 587 genes implicated in energy metabolism were obtained after eliminating duplicate genes. Among them, 3 genes were excluded because of no related record in *TCGA* database, and 12 genes with FPKM = 0 in 50% of samples were also excluded. Finally, 572 genes were enrolled as candidate genes for subsequent analysis. The molecular subtypes were constructed based on these prognostic genes using cumulative distribution function (CDF) method, and the optimal number of subtypes were determined according to the CDF Delta area.

**Table 2:** Pathways associated with energy metabolism in the Reactome pathway database

Metabolic pathways from Reactome	Pathway ID	Gene Count
Biological oxidations	R-HSA-211859	216
Metabolism of carbohydrates	R-HSA-71387	290
Mitochondrial Fatty Acid Beta-Oxidation	R-HSA-77289	37
Glycogen synthesis	R-HSA-3322077	16
Glycogen metabolism	R-HSA-8982491	27
Glucose metabolism	R-HSA-70326	90
Glycogen breakdown (glycogenolysis)	R-HSA-70221	15
Glycolysis	R-HSA-70171	71
Pyruvate metabolism	R-HSA-70268	31
Pyruvate metabolism and Citric Acid (TCA) cycle	R-HSA-71406	55
Citric acid cycle (TCA cycle)	R-HSA-71403	22
Sum	871(unique:587)	

### Evaluation of immune characteristics between molecular subtypes

The distribution of tumor-infiltration immune cells, ImmuneScore, StromalScore and ESTIMATEScore between the two subtypes were evaluated as previously described [17]. The ImmuneScore, StromalScore and ESTIMATEScore are represent the relative proportion of immune cells, stromal cells and the purity of tumor tissues, respectively.

### Identification of co-expression genes by weighted gene correlation network analysis

Co-expressed genes and modules were detecting by using the weighted gene correlation network analysis (WGCNA) co-expression algorithm as previously described [17] with the soft-threshold power *β* set to 8.

### Screening of robust prognostic feature genes and construction of gene signature

To narrow the gene range and maximize the accuracy, LASSO Cox regression analysis [18], a method screening signatures with generally effective prognostication performance by performing automatic feature selection, was performed by using the R package glmnet, and optimal genes were evaluated by 3-fold cross validation. Multivariate Cox regression survival analysis was performed to construct the prognostic risk model. Risk score for each patient in the training set was calculated with the linear combinational of the signature gene expression weighting by their regression coefficients. Risk score = (expr_gene1_ x coefficient_gene1_) + (expr_gene2_ x coefficient_gene2_) + … + (expr_genen_ x coefficient_genen_). Receiver operating characteristics (ROC) curves, carried out by using the R package timeROC, was used to analyze the sensitivity and specificity of the gene signature for prognostication of OS. Then the risk-score was Z-scored, and zero was selected as the threshold. The optimal cut-off value of the expression level of these eight genes were determined by R package maxstat.

### Bioinformatic analysis

Functional enrichment analysis was applied for identifying relationship between the molecular subtypes and biological functions using the R package GSVA. The classical gene sets of Kyoto Encyclopedia of Genes and Genomes (KEGG) pathways (c2.cp.kegg.v11.0.symbols) were measured to decipher the phenotype. *P* value < 0.05 was considered as the FDR cutoff value. The open source R WGCNA version 1.68 was used for WGCNA analysis and visualization, and the open source R GOplot version 1.3 was used for the visualization of enrichment functional integration network.

### Statistical analysis

Kaplan-Meier curves were applied to evaluate the difference on OS between different groups. Univariate and multivariate Cox regression analyses were used to assess the independent prognosis predictive factors. To predict the survival rate of patients, nomogram, a statistical method that can present the influence of each variable on the outcome using the length of a straight line, and the effect of different values of each variable on the outcome [19], was established using the R package RMS. The decision curve analysis (DCA) [20] was performed to assess the clinical usefulness of the nomogram in comparison with the gene signature and clinicopathological parameters. The regression model was established using the CPH function of R package RMS, and the Nomogram function of R package RMS was used to construct the nomogram. The DCA analysis was performed and visualized using the DECISIon_curve function of R package RMDA. All statistical analyses were performed using R 3.6.0 (https://mirrors.tuna.tsinghua.edu.cn/CRAN/) with default software parameters. *P* value < 0.05 was considered significant statistically.

## Results

### Identification of molecular subtypes in ESC

**Fig. 1:**
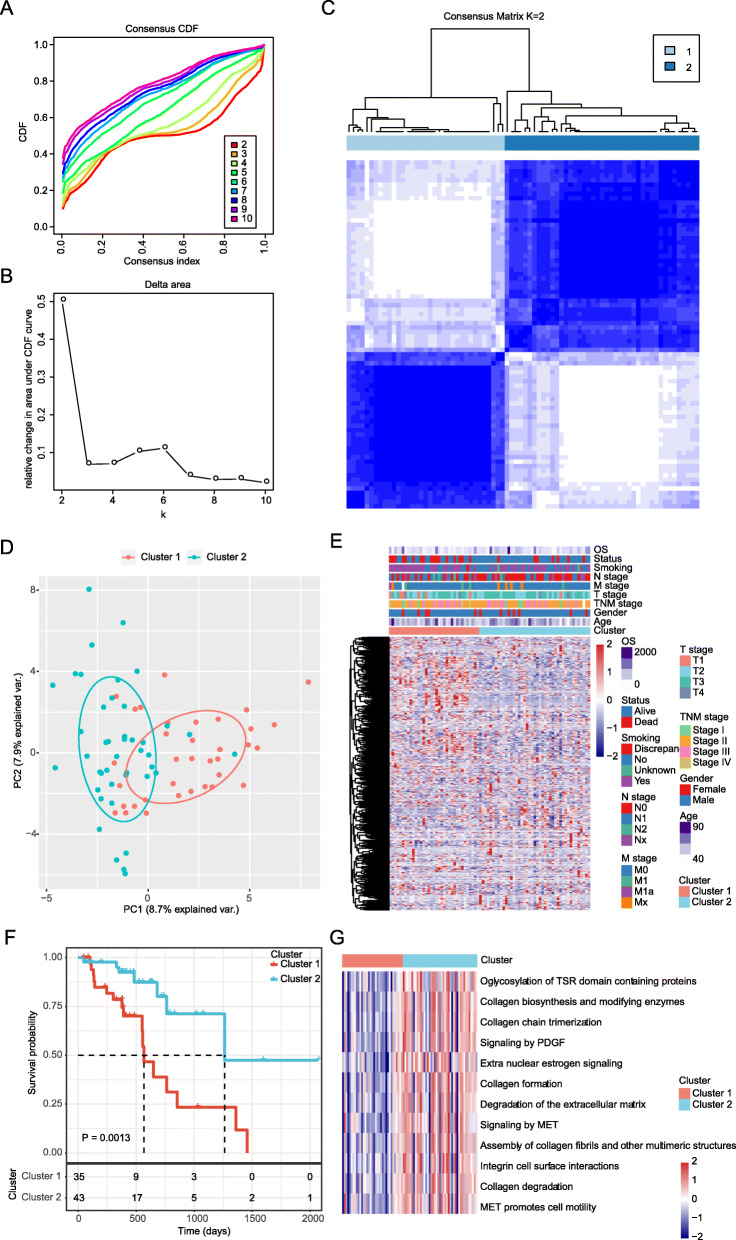
Energy metabolism related molecular subtypes in ESC. **a**. cumulative distribution function (CDF) curve; **b**. CDF Delta area curve, Delta area curve of consensus clustering, indicating the relative change in area under the cumulative distribution function (CDF) curve for each category number k compared with k - 1. The horizontal axis represents the category number k and the vertical axis represents the relative change in area under CDF curve. **c**. Heat map of samples (consensus K = 2); **d**: PCA analysis of the expression profile of EMRGs and scatter plots of the first two principal components; **e**. Heat map of EMRGs expressions in the two molecular subtypes. **f**. Kaplan-Meier curve on the differences of prognosis between the two subtypes; **g**. Pathway outcomes of differences on KEGG pathway scores between the two subtypes. Functional enrichment analysis was conducted using R package GSVA

By univariate Cox regression survival analysis, 43 EMRGs were correlated and identified with the OS of patients with ESC in the *TCGA* dataset (Supplementary Table S1). Then, by consensus unsupervised clustering of 78 samples from patients with ESC using the 572 EMRGs, two clusters were found had lower values of ambiguously clustered pairs (PAC), which reflected the near-perfect stability of the samples under the correct K value distribution (Fig. 1a-b). The relative change of the area under the CDF curve reveals a nearly perfect stable distribution of the samples starting from 2 clusters (Fig. 1c). Principal component analysis (PCA, Fig. 1d) and consensus heatmaps (Fig. 1e) also showed a fairly stable distribution samples in the 2 clusters. After evaluating the relative changes in the area under the CDF curve, PAC value, PCA and consensus heatmaps, we chose a two-cluster solution. Thus, two molecular subtypes were constructed based on these 43 prognostic EMRGs.

In the Kaplan–Meier curve with log-rank tests, patients in Cluster 1 showed worse overall survival (OS) time than that in Cluster 2 (Fig. 1f). By analyzing the KEGG pathways, 12 pathways, such as O glycosylation of TSR domain containing proteins, PDGF signaling and extra nuclear estrogen signaling, were found significantly different between the two subtypes (Fig. 1g). However, no significantly difference on clinicopathological feature was found between the two molecular subtypes in patients with ESC (Supplementary Figure 1). In addition, the differences on immune characteristics between the two subtypes were analyzed. Although there was no significant difference on the immune infiltration between the two subtypes, Cluster 2 showed the higher relative proportion of stromal cells than Cluster 1 (Supplementary Figure 2).

### Determination of hub genes by WGCNA analysis

**Fig. 2:**
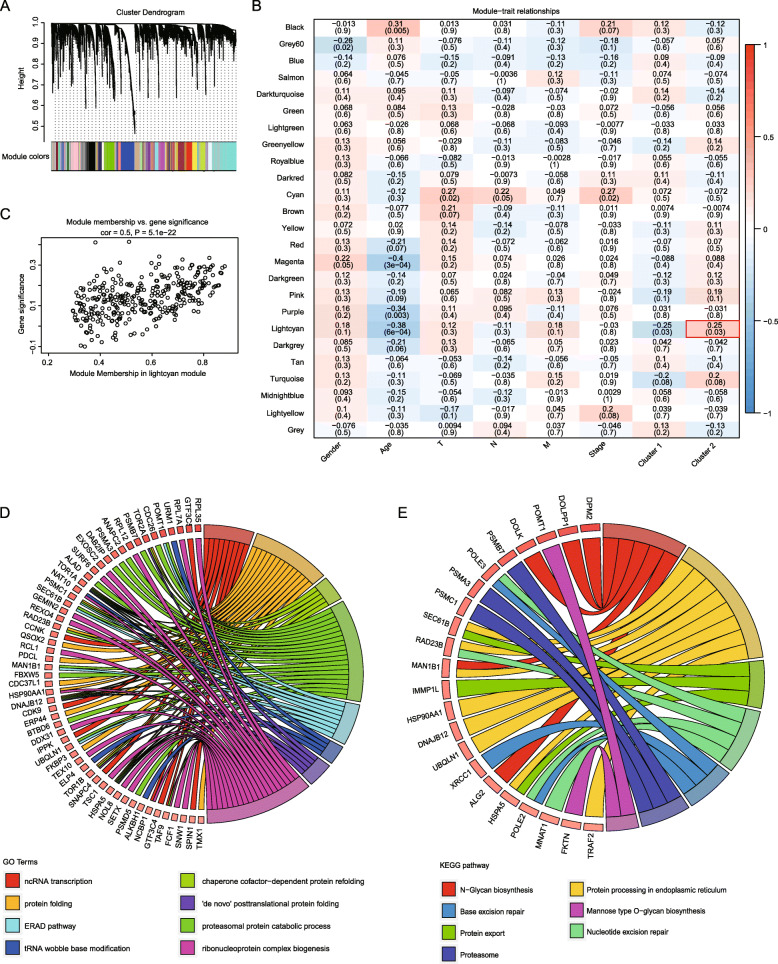
Hub genes identified by WGCNA co-expression analysis. **a**. Gene dendrogram and module colors; **b**. Relationship between the 25 modules and the clinical phenotypes and molecular subtypes. **c**. The correlation of lightcyan module with Cluster 2 in the TCGA ESC dataset; **d**. GO BP enrichment functional integration network developed by genes in the lightcyan module; **b**: KEGG enrichment functional integration network developed by genes in the lightcyan module. The open source R WGCNA version 1.68 was used for WGCNA analysis and visualization, and the open source R GOplot version 1.3 was used for the visualization of enrichment functional integration network

By module fusion based on the expression profile of EMRGs in the *TCGA* ESC dataset, 25 co-expression modules were obtained (Fig. 2a), among them grey modules represent gene sets couldn’t be fused. By analyzed the relationship between the identified modules and clinical characteristics as well as molecular classifications, we found that Cluster 2 was significantly correlated with the lightcyan module, which including 326 genes (*r* > 0.4, *P* < 0.05), whereas there was no module significantly correlated with Cluster 1 (Fig. 2b). In addition, genes in the lightcyan module were largely associated with the Cluster 2 subtype (Fig. 2c). Therefore, the lightcyan module which is closely related to energy metabolism-based subtype of ESC was considered as the hub module, and genes involved in this module were regarded as hub genes. Besides, functional enrichment analysis demonstrated that these 326 hub genes were significantly (FDR < 0.01) involved in 290 GO biological process categories (e.g., ERAD pathway and ncRNA transcription, Fig. 2d, Supplementary Table S2) and 7 KEGG pathways (e.g., N-Glycan biosynthesis and protein export, Fig. 2e, Supplementary Table S3).

### Identification of EMRG signature associated with overall survival in patients with ESC

**Fig. 3:**
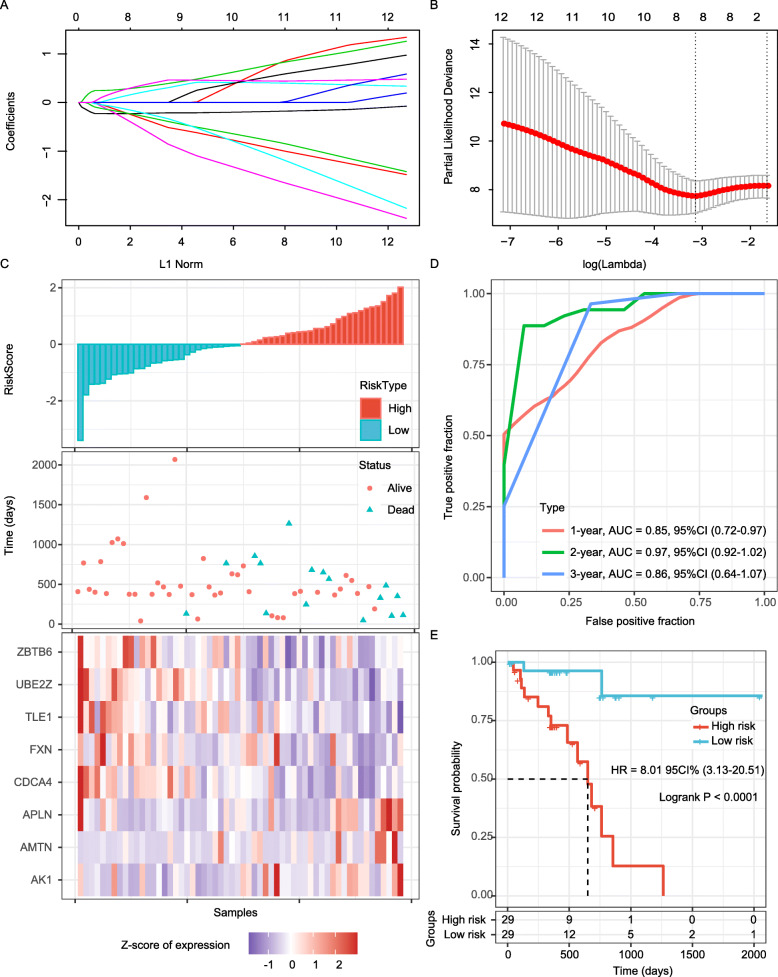
Evaluation of the performance of the 8-gene signature in the training dataset. **a**. Trajectory change of each independent variable. The X axis represents the log value of the independent variable lambda; the Y axis represents the coefficient of the independent variable. **b**. Confidence intervals of lambda. **c**. Risk score, survival time, survival status and expression of the 8-gene signature in the training set. **d**. ROC curve of the 8-gene signature for 1-year, 3-year and 5-year survival in the training set. **e**. Kaplan-Meier survival curve based on the 8-gene signature in the training set. ROC, receiver operating characteristic; AUC, area under the curve; HR, hazard ratio; CI, confidence interval

Patients diagnosed with ESC from *TCGA* database (*n* = 78) was enrolled in the establishment of gene signature, and 75% of them were randomly assigned to the training set (Table 1). To identify novel genes associated with the clinical outcome of patients with ESC, univariate Cox proportional hazard regression was applied to analyze those 326 hub genes. And then genes significantly associated with OS (*P* < 0.05) were entered into dimensional-reduction analysis by performing LASSO regression analysis. Finally, eight independent prognostic genes (including UBE2Z, AMTN, AK1, CDCA4, TLE1, FXN, ZBTB6 and APLN) were confirmed (*P* < 0.05, Table 3) with 3-fold cross-validation and minimized error rate λ = 0.043 (Fig. 3a-b). By applied Kaplan-Meier analysis on these eight genes using the optimal cut-off value of the expression level of each gene, all eight genes were conformed significant associated with OS (Supplementary Figure 3). Among them, AMTN, AK1 and APLN were significant negative correlation with OS, while UBE2Z, CDCA4, TLE1, FXN and ZBTB6 were significant positive correlated with OS in the training set. The final 8-gene signature was calculated using multivariate Cox survival analysis, and a EMRG-based prognostic gene signature was established to calculate the survival risk of each patient as follows: RiskScore = − 0.223*exp^UBE2Z^ + 0.323*exp^AMTN^ + 0.220*exp^AK1^ - 0.572*exp^CDCA4^ - 0.350*exp^TLE1^ - 0.291*exp^FXN^ - 0.223*exp^ZBTB6^ + 0.366*exp^APLN^.

**Table 3:** Univariate Cox regression analysis result of 8 genes in *TCGA* training set

Gene	*P* value	Harzard ratio	Low 95%CI	High 95%CI	Coefficient
UBE2Z	0.024	0.448	0.223	0.898	−0.223
AMTN	0.007	1.931	1.198	3.112	0.323
AK1	0.038	1.822	1.033	3.215	0.220
CDCA4	0.016	0.381	0.173	0.836	−0.572
TLE1	0.039	0.458	0.218	0.963	−0.350
FXN	0.040	0.408	0.173	0.961	−0.291
ZBTB6	0.040	0.519	0.278	0.972	−0.223
APLN	0.039	1.661	1.026	2.690	0.366

According on the risk score formula, patients were classified into high-risk or low-risk group (Fig. 3c). The heatmap which shown the expression profile of the eight genes illustrated that as the risk score of patients increased, the expression of prognosis-risky genes (AMTN, AK1 and APLN) were distinctly upregulated; in contrast, the expression of prognosis-protective genes (UBE2Z, CDCA4, TLE1, FXN and ZBTB6) were downregulated (Fig. 3c). The accuracy of the prognostic 8-gene signature for 1-year, 3-year and 5-year survival, as reflected by the ROC curve, was 0.85, 0.97 and 0.85, respectively (Fig. 3d). Finally, we classified samples with Zscore-based Riskscore > 0 into the high-risk group, and the others into the low-risk group. Kaplan-Meier curve analysis revealed that the OS time of patients in the low-risk group was significantly longer than that in the high-risk group (*P* < 0.0001; Fig. 3e). Considering that the heatmap may be affected by outliers and show insignificant results, we evaluated the expression differences of these 8 genes between the high-risk and low-risk groups. As expected, these genes showed significant different expression levels between the two groups (Supplementary Figure 4A).

To further analyze the significantly KEGG pathway the 8-gene signature may involve in, the enrichment score of KEGG pathway in each sample was calculated. It was found that the 8-gene signature was significantly related to three pathways: VEGF signaling pathway, NOTCH signaling pathway, and neurotrophin signaling pathway (FDR < 0.05; *r* > 0.35; Supplementary Figure 4b).

### Validation of the 8-gene signature in the entire *TCGA* dataset and two GEO datasets

**Fig. 4:**
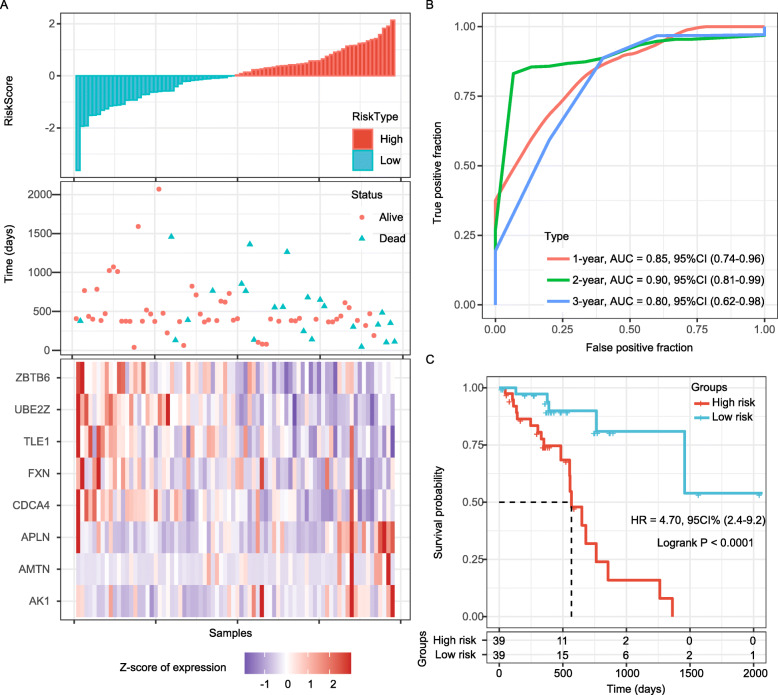
Internal validation of the 8-gene signature’s robustness in the *TCGA* cohort. **a**. Risk score, survival time, survival status and expression of the 8-gene signature in the internal validation cohort. **b**. ROC curve of the 8-gene signature for 1-year, 3-year and 5-year survival in the internal validation. **c**. Kaplan-Meier survival curve based on the 8-gene signature in the internal validation set. ROC, receiver operating characteristic; AUC, area under the curve; HR, hazard ratio; CI, confidence interval

The entire *TCGA* ESC dataset was used for internal validation, and the risk score of each sample was computed, which showed that the association between the gene expression and risk score was consistent with the training set (Fig. 4a). The ROC curve displayed that the accuracy of the prognostic 8-gene signature for 1-year, 3-year and 5-year survival was 0.85, 0.90 and 0.80, respectively (Fig. 4b). Patients in the internal validation dataset were divided into high-risk and low-risk groups with the same cutoff value as used in the training set. As expected, patients in the validation set with low risk-scores had longer OS than those with high risk scores (*P* < 0.0001; Fig. 4c).

**Fig. 5:**
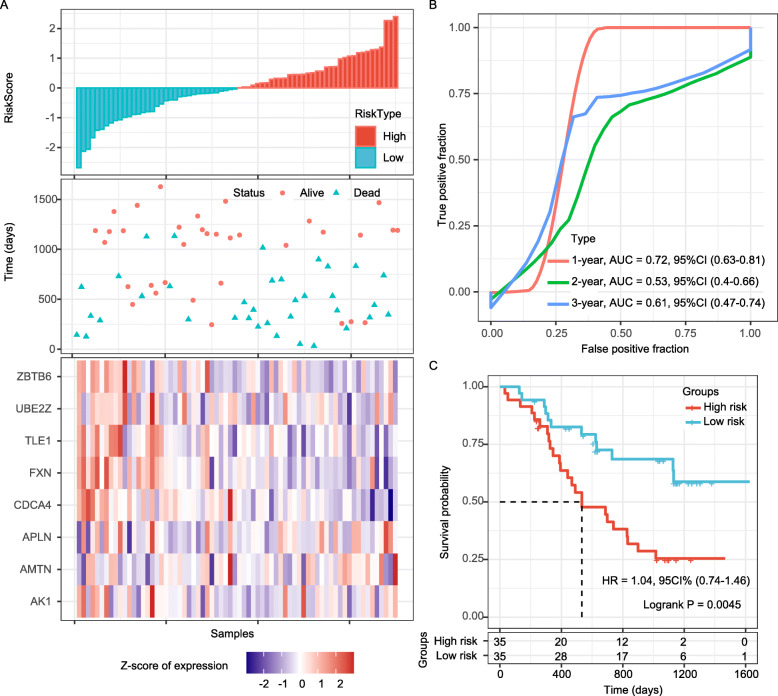
External validation of the 8-gene signature’s robustness in the GSE54993 cohort. **a**. Risk score, survival time, survival status and expression of the 8-gene signature. **b**. ROC curve of the 8-gene signature for 1-year, 3-year and 5-year survival. **c**. Kaplan-Meier survival curve based on the 8-gene signature. ROC, receiver operating characteristic; AUC, area under the curve; HR, hazard ratio; CI, confidence interval

**Fig. 6:**
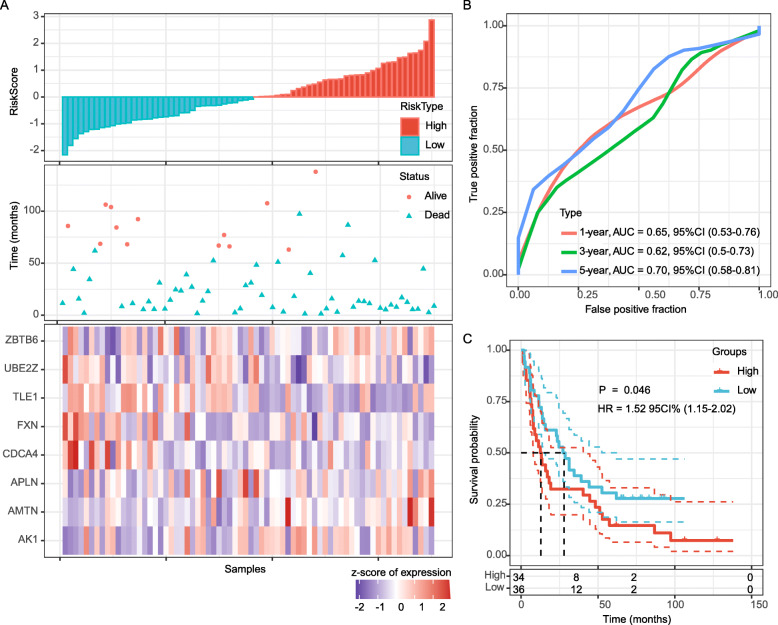
External validation of the 8-gene signature’s robustness in the GSE19417 cohort. **a**. Risk score, survival time, survival status and expression of the 8-gene signature. **b**. ROC curve of the 8-gene signature for 1-year, 3-year and 5-year survival. **c**. Kaplan-Meier survival curve based on the 8-gene signature. ROC, receiver operating characteristic; AUC, area under the curve; HR, hazard ratio; CI, confidence interval

The prognostication efficiency of our 8-gene signature was also calculated in the external validation datasets (GSE54993 and GSE19417), both contains 70 patients with definite diagnosis of ESC and prognostic information (Fig. 5a and Fig. 6a). In the GSE54993 dataset, the ROC curve displayed that the accuracy of the prognostic 8-gene signature for 1-year, 3-year and 5-year survival was 0.72, 0.53 and 0.61, respectively (Fig. 5b). In addition, patients in the GSE54993 validation sets with high risk-scores had shorter OS than those with low risk scores (*P* = 0.0045; Fig. 5c). In the GSE19417 dataset, the ROC curve displayed that the accuracy of the prognostic 8-gene signature for 1-year, 3-year and 5-year survival was 0.62, 0.62 and 0.70, respectively (Fig. 6b); Patients in low risk-score group had longer OS than those in the high-risk group (*P* = 0.046; Fig. 6c), of which 34 samples were classified as high-risk and 36 samples were classified as low-risk. Therefore, the 8-gene signature exhibited steady effective prognostication performance in the internal and external validation sets.

Given that the *TCGA* ESC dataset also include the disease specific survival (DFS) information of patient, the correlation of our 8-gene signature was further analyzed using the *TCGA* cohort. The ROC curve displayed that the accuracy of the prognostic 8-gene signature for 1-year, 2-year and 3-year survival were all > 0.80 (Supplementary Figure 5A). Patients in the high risk-score group (*n* = 29) had significant shorter DFS than those in the low risk-score group (n = 29, *P* = 0.0011, Supplementary Figure 5B).

### Cox regression analyses of the 8-gene signature

**Fig. 7:**
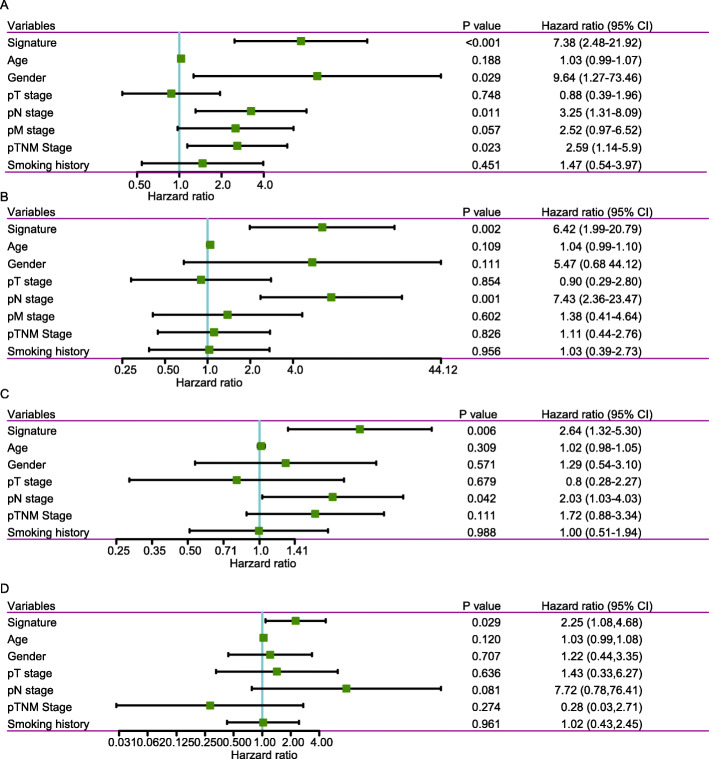
Univariate and multivariate Cox regression analyses of the 8-gene signature. **a**. Forest plot of the univariate Cox regression analyses in *TCGA* ESC dataset. **b**. Forest plot of the multivariate Cox regression analyses in *TCGA* ESC dataset. **c**. Forest plot of the univariate Cox regression analyses in GSE54993 dataset. **d**. Forest plot of the multivariate Cox regression analyses in GSE54993 dataset

To identify the independence of the 8-gene signature in clinical application, its prognostic value in the *TCGA* cohort was analyzed by using univariate and multivariate Cox regression analysis. Clinicopathological parameters including the 8-gene signature, age, gender, pathological T stage, N stage, M stage and TNM stage were included in the analysis. The univariate analysis showed that gender, N stage, tumor stage, and the 8-gene signature were significantly related to the OS in ESC (all *P* < 0.05; Fig. 7a). The multivariate analysis showed that only N stage (HR = 7.43, 95%CI = 2.36–23.47, *P* = 0.001) and the 8-gene signature (HR = 6.42,95%CI = 1.99–20.79, *P* = 0.002) were independent prognostic factors in ESC (Fig. 7b). In GSE54993 dataset, univariate analysis results showed that N stage and the 8-gene signature were significantly related to the OS in ESC (all *P* < 0.05; Fig. 7c). However, the multivariate analysis showed that only the 8-gene signature (HR = 2.25,95%CI = 1.08–4.68 *P* = 0.029) was an independent prognostic factor in ESC (Fig. 7d).

### Development of nomograms to predict the outcome of patients with ESC

**Fig. 8:**
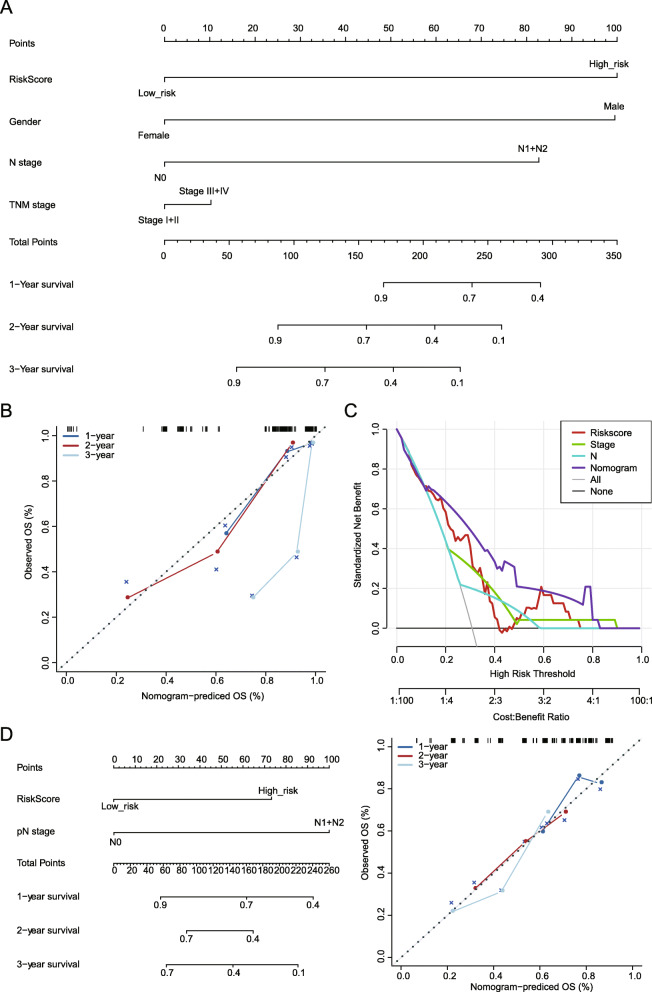
Nomograms for prediction of the outcome of patients with ESC. **a**: Nomogram developed by integrating the signature risk-score with the clinicopathologic features in the *TCGA* ESC dataset. **b**. Calibration curves of nomogram for predicting OS at 1-year, 3-year and 5-year in the *TCGA* ESC dataset. **c**. DCA plots developed by integrating the signature risk-score with the clinicopathologic features in the *TCGA* ESC dataset. **d**. Nomogram developed by integrating the signature risk-score with the clinicopathologic features in the GSE54993 dataset. **f**. Calibration curves of nomogram for predicting OS at 1-year, 3-year and 5-year in the GSE54993 dataset. The Nomogram function of R package RMS was used to construct the nomogram. The DCA analysis was performed and visualized using the DECISIon_curve function of R package RMDA

A nomogram was conducted using the *TCGA* dataset based on the 8-gene signature and all clinicopathological parameters (Fig. 8a). By scoring the predictors, the higher the total score, the shorter the survival time. The calibration curves for the probabilities of 1-, 3-, and 5-year OS indicated an excellent agreement between the nomogram prediction and observed outcomes in the *TCGA* dataset (Fig. 8b). A decision curve analysis (DCA) was also applied to evaluate the 8-gene signature with these clinicopathological parameters, in which the curve of TNM stage and N stage are very close to the two extreme curves, which means that them has less clinical application value (Fig. 8c). However, the risk-score and the established nomogram presented a higher net benefit together with broader range of threshold probability than TNM stage and N stage. And the nomogram is better than the 8-gene signature (Fig. 8c). In addition, the nomogram and calibration curves for the probabilities of 1-, 3-, and 5-year OS in the GSE54993 dataset also indicated an excellent agreement between the nomogram prediction and observed outcomes (Fig. 7d-e).

### The performance of the 8-gene signature in comparison to previous signatures in *TCGA* ESC dataset

**Fig. 9:**
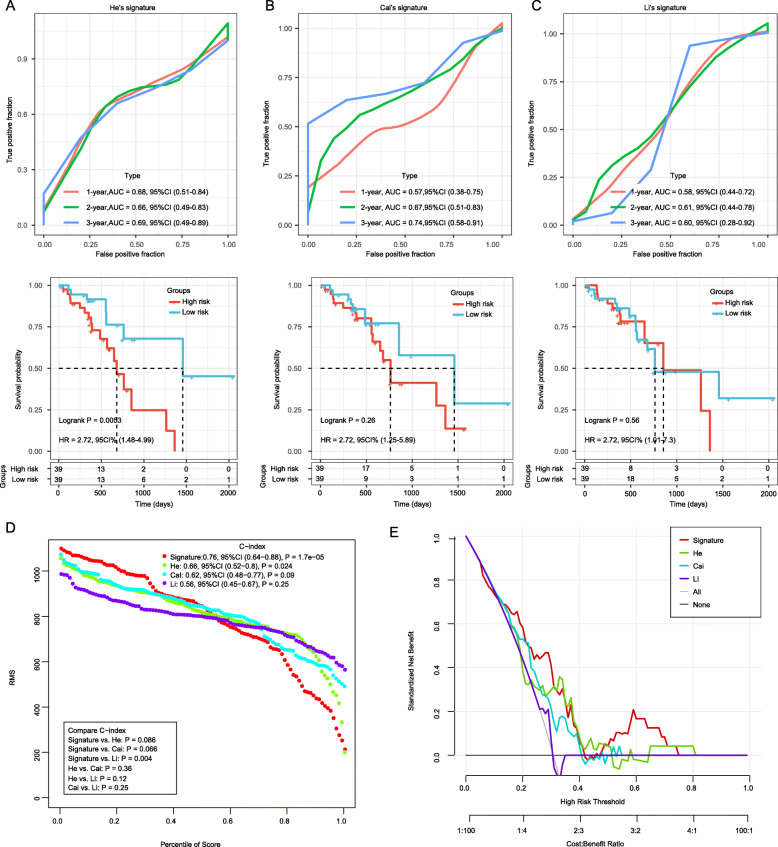
The performance of the 8-gene signature in comparison to previous signature in *TCGA* ESC dataset. **a**: ROC curve and Kaplan-Meier survival analysis of the 5-gene signature of He et al.; **b**: ROC curve and Kaplan-Meier survival analysis of the 3-gene signature of Cai et al.; **c**: ROC curve and Kaplan-Meier survival analysis of the 3-gene signature of Li et al.; **d**: Restricted mean survival time (RMST) curve developed by integrating the indicated 4 signatures; **e**: DCA plots developed by integrating the indicated 4 signatures. The regression model was established using the CPH function of R package RMS

To assess the predictive power of the 8-gene signature, the performance of three previous reported robust prognostic risk models were enrolled for comparison, including a 5-gene signature developed by He et al. [21], a 8-gene signature developed by Cai et al. [22] and a 9-gene signature developed by Li and colleagues [23]. The risk-score of each ESC sample in *TCGA* cohort was calculated according to the corresponding genes in all three models by applying the same method being reported [21–23]. The ROC of each gene signature was evaluated, and the area under the curve of all three models were larger than 0.6 (Fig. 9a-c upper). Kaplan-Meier curve analysis revealed that only He’s 5-gene signature showed significant prognostic value in predicting OS (*P* = 0.0053; Fig. 9a down). Restricted mean survival time (RMST) was applied to calculated and compared the C-index of all four signatures. Although the C-index of our 8-gene signature was only significantly higher than that of Li’s 3-gene signature (*P* = 0.0041), our 8-gene signature showed the highest C-index (0.76; Fig. 9d). We also applied a DCA to evaluate the 8-gene signature with these three signatures, in which the curve of Li’s 3-gene signature is very close to the two extreme curves, while the net benefit and broader range of threshold probability of the 8-gene signature ranked as the highest one (Fig. 9e), indicating that the 8-gene signature in the present study exhibited a best prognostication performance. Taken together, these results imply that this signature is more suitable for predicting the prognosis of patients with ESC in clinical practice.

## Discussion

Metabolic remolding is a hallmark of cancer [7]. Now, the essence of energy metabolism has spread to the field of ESC cells [5, 6]. However, in ESC, the relationship of energy metabolism-based gene with the prognosis and their potential impact on biological behaviors have not been elucidated yet. In this study, we established and validated a novel prognostic gene signature based on energy metabolism related eight-gene to improve the prediction of overall survival (OS) after surgery for patients with ESC. Our results showed that this energy metabolism gene signature can effectively categories patients into high-risk groups and low-risk groups with obviously differences on 1-, 3- and 5-year OS. Furthermore, the performance of this proposed gene signature on prognostication of ESC is significantly better than the other clinicopathological risk parameters.

ESC is associated with high morbidity and mortality rates [24]. Postoperative chemotherapy regimens for patients at advance stage including platinum combined or fluorouracil-based regimens and sometimes anthracyclines, all of which exhibit considerable cytotoxic effects. However, currently there is still lack of recurrence risk prediction solution for postoperative management of ESC. To mitigate the currently limited therapeutic options, it is necessary to detect the optimized biomarker for the prognostication of patients, which will shed new light on the target therapy of ESC. Traditional clinicopathological risk parameters can’t clearly distinguish the high-risk and low-risk patients with ESC. Despite recent advances in recognizing genomic drivers of ESC [4], seldom prognostic markers are clinically available in ESC. Multiple genes have been implicated in the regulation of cell energy metabolism process in ESC [5, 6, 12, 25, 26]. In particular, several EMRGs, such as RAC1 [5], TKTL1 [27], and PFKFB3 [28] were shown to be associated with prognosis in patients with ESC. However, previous studies were limited by just single gene detected, small sample sizes, and lack of independent validation. The use of the LASSO Cox regression model [18] and nomogram [19] allowed us to combine multiple gene into one signature, which has significantly better prognostic accuracy than that of single gene alone.

Numerous previous studies also identified gene signature for the prognostication of ESC. For example, He et al. [21] identified a 5-gene model which contained RFC2, DDIT3, CXCL8, ELL2 and RAB27A. Cai et al. [22] proposed a signature consisting of 8 lymph node metastasis related genes (CDK5R2, CSH2, CA7, SPANXN5, NR5A1, CRP, NOTUM, and KRTAP71), which shared no same gene with He’s gene-signature. Li et al. [23] discovered another gene signature by enrolled the gene expression level of nine immune related gene including CD38, HMGB1, ICOSLG, ABL1, ATF2, ATG5, C6, IL12RB2 and PLAU, that was totally different from the previous two modules. Given the difference on target gene subsets used for screening prognostic genes, there was no overlapping genes among our gene list and these three gene sets. We further compared the predictive performance of the present signature with that of the three previous signatures. It was confirmed that among these signatures, the eight-gene model had the highest AUC and C-index values. Our results revealed that the EMRG to some extents outperforms molecules involved in other cancer relevant pathways in the prognostication of patients with ESC. In addition, when focus on the eight genes in our gene signature, CDCA4 gene with the largest absolute value of coefficient maybe the one that contributed most to the signature.

Some limitations of this study should be taken into consideration. Although *TCGA* and GSE54993 dataset enrolled both Caucasian and Chinese population, majority of ESC was occurred among Eastern Asian and Eastern & Southern African population, the distribution of clinicopathological characteristics might be different in other areas we didn’t included, making it susceptible to the inherent biases of such a study design. Undoubtedly, our results should be further validated in cohort from some worldwide multicenter. In addition, genes play versatile biological and pathological roles in cancer cells are always associated with the intrinsic characteristics of tumor, and thus can be specific predictor of cancer progression and prognosis [10–13]. However, the biofunction of our eight genes have not yet been investigated in previous studies, and the role of these genes should be further systematically explored according to the actual diseased tissues or even cell lines and animal models. When focus on these eight genes, CDCA4 gene with the largest absolute value of coefficient maybe the one contributed most to the signature, therefore, CDCA4 should be primary concerned in future research.

## Conclusions

In summary, our findings show that the novel eight-gene signature provides prognostication value that complements clinicopathological risk parameters, and more accurately predicts overall survival for patients with ESC than clinicopathological risk factors alone, as well as three previous reported prognostic model. This gene signature might, therefore, help with patient risk stratification and precise management of patients with ESC.

## Supplementary Information

**Additional file 1: Supplement Figure S1.** Distribution of clinicopathological parameters in the two subtypes.

**Additional file 2: Supplement Figure S2.** The proportions of B cell, CD4+T cell, CD8+T cell, Neutrophil, Macrophage, Dendritic cell (DC), ImmuneScore, StromalScore, and ESTIMATEScore between the two subtypes. The enumeration of six tumor-infiltration immune cells was estimated using the “Tumor Immune Estimation Resource” (TIMER, https://cistrome.shinyapps.io/timer/) tool.

**Additional file 3: Supplement Figure S3.** Kaplan-Meier analysis on the eight genes using the optimal cut-off value of the expression level of each gene.

**Additional file 4: Supplement Figure S4.** A. the differences on the expression level of these 8 gene between the two groups; B. single sample GSEA analysis results of pathways significantly associated with the 8-gene signature.

**Additional file 5: Supplement Figure S5.** A. ROC curve of the 8-gene signature for 1-year, 3-year and 5-year DFS; B. Kaplan-Meier survival curve of DFS based on the 8-gene signature. ROC, receiver operating characteristic; AUC, area under the curve; HR, hazard ratio; CI, confidence interval.

**Additional file 6: Supplementary** **Table S1.** Prognostic value of 43 EMRGs in TCGA dataset.

**Additional file 7: Supplementary** **Table S2.** GO Biological process clustered by 326 hub genes.

**Additional file 8: Supplementary** **Table S3.** KEGG pathways clustered by 326 hub genes.

## Data Availability

The datasets generated and analyzed during the current study are available in the TCGA repository (https://portal.gdc.cancer.gov/) with the accession number TCGA-ESCA, and the GEO repository (https://www.ncbi.nlm.nih.gov/geo/) the accession number were GSE54993 and GSE19417. Coherently expressed signatures of human metabolism-related pathways were all download from the Reactome pathway database (https://reactome.org/) and derived by aggregating MSigDB version 7.0 gene sets.

## Abbreviations

ESC: Esophagus carcinoma
EMRG: Energy metabolism-related genes
OS: Overall survival
LASSO: Least absolute shrinkage and selection operator
ROC: Receiver operating characteristic
*TCGA*: The Cancer Genome Atlas
DCA: Decision curve analysis
EMST: Restricted mean survival time
FPKM: Fragments Per Kilobase of exon model per Million mapped fragments
CDF: Cumulative distribution function
ESTIMATE: Estimation of STromal and Immune cells in MAlignant Tumours using Expression data
WGCNA: Weighted gene correlation network analysis
TOM: Topology overlapping matrix
KEGG: Kyoto Encyclopedia of Genes and Genomes
PCA: Principal component analysis
DFS: Disease specific survival
