# Supplementary figures and images for: An energy metabolism-based eight-gene signature correlates with the clinical outcome of esophagus carcinoma
Source: BMC Cancer. 2021 Apr 1;21:345. doi: 10.1186/s12885-021-08030-0 (PMC8015196; doi:10.1186/s12885-021-08030-0)

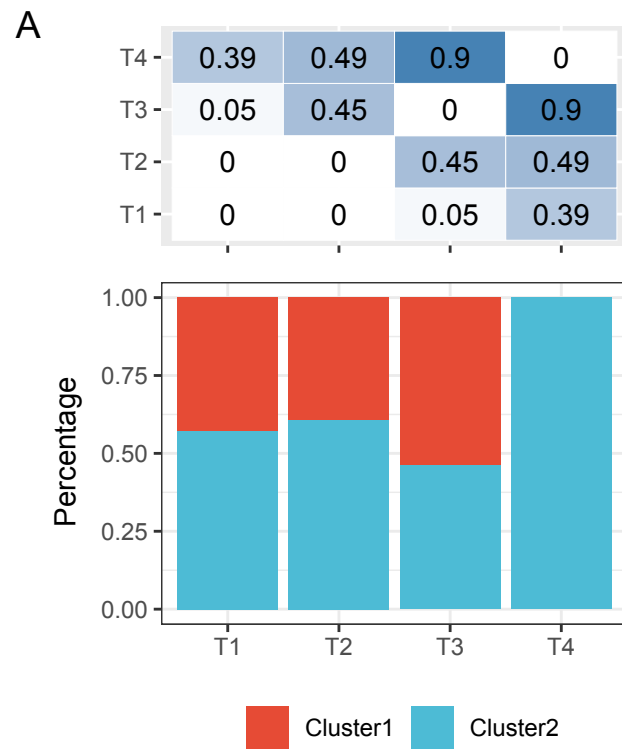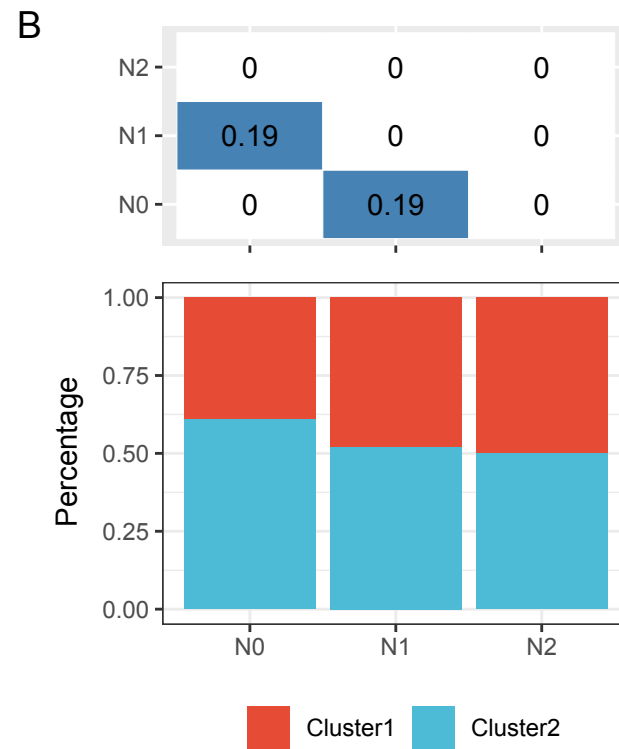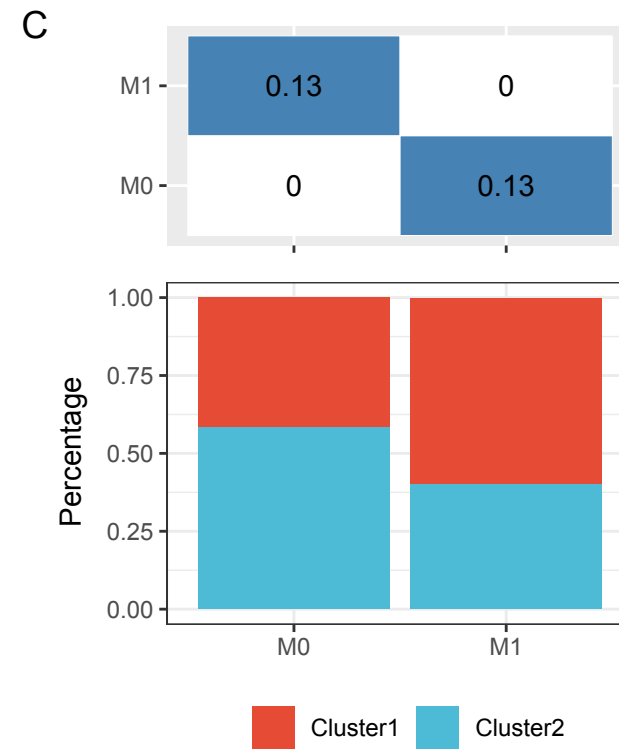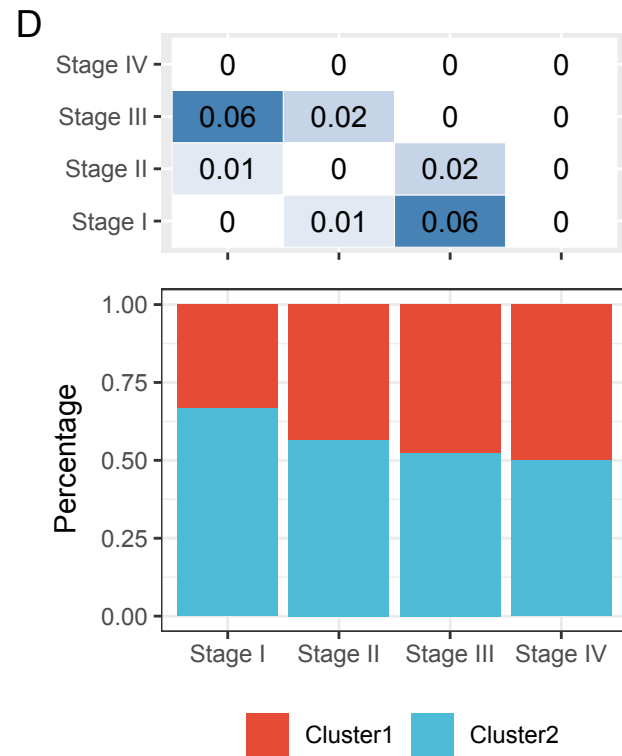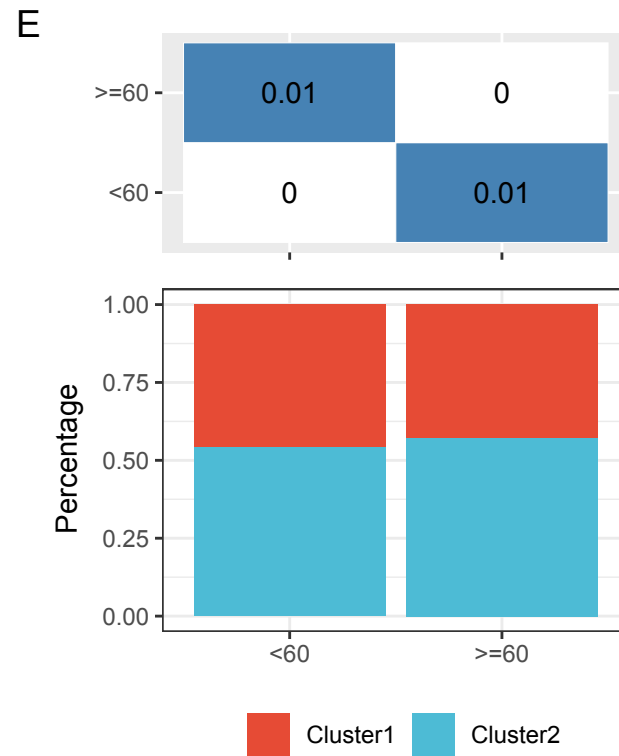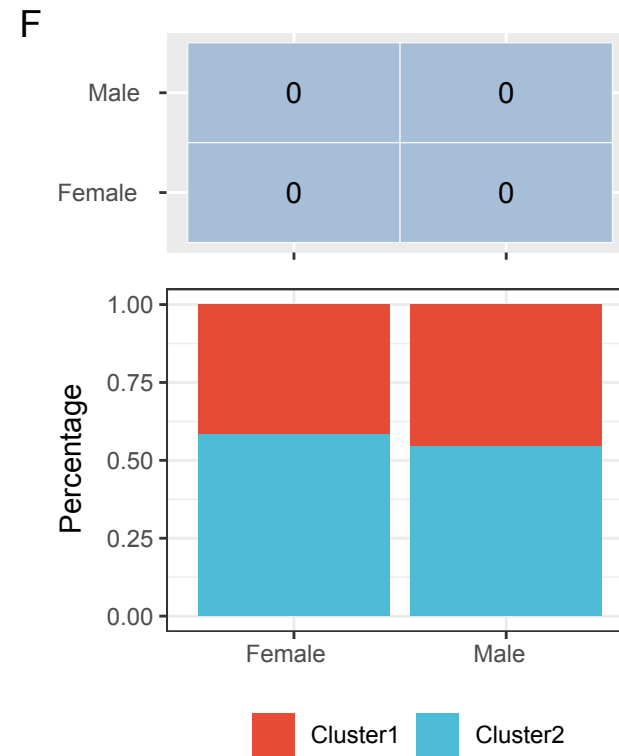

Supplement: Supplementary file 1 — Additional file 1: Supplement Figure S1. Distribution of clinicopathological parameters in the two subtypes. [file 12885_2021_8030_MOESM1_ESM.pdf]

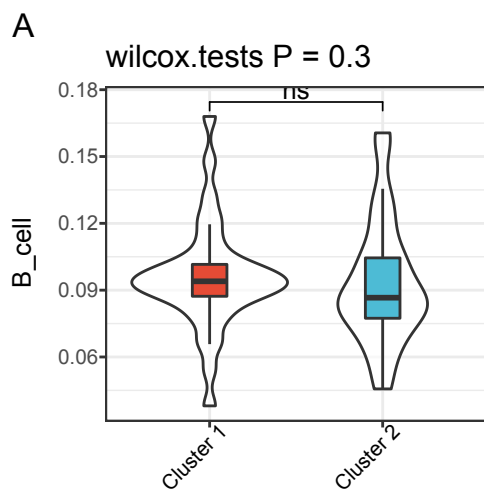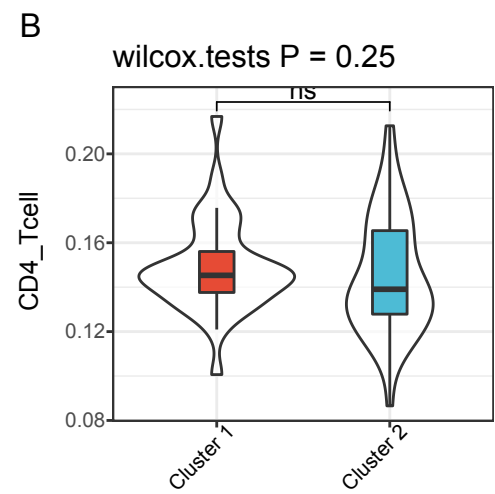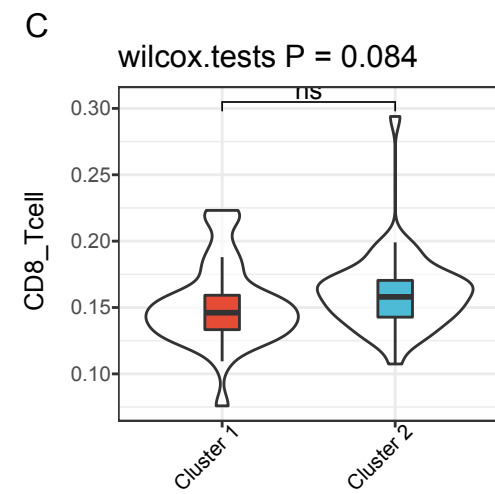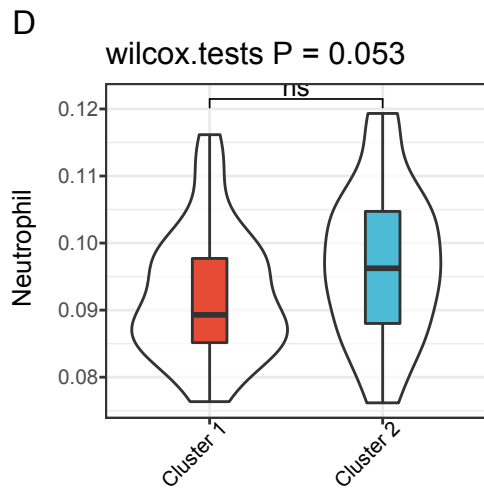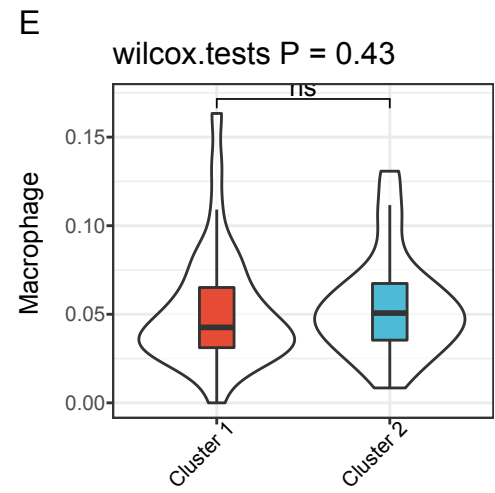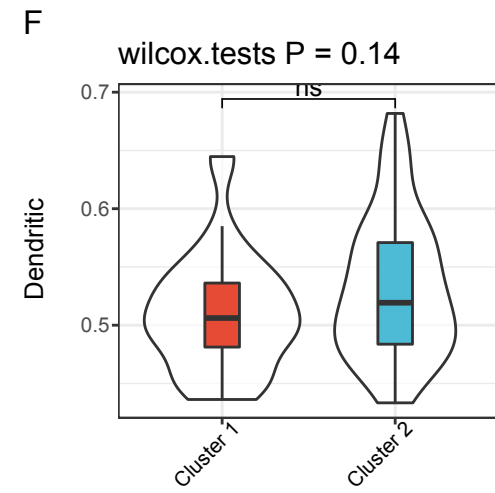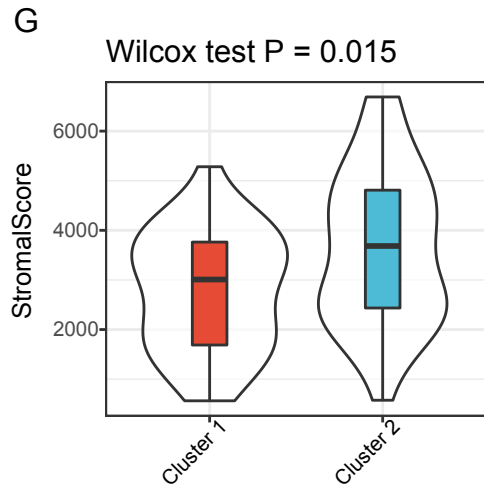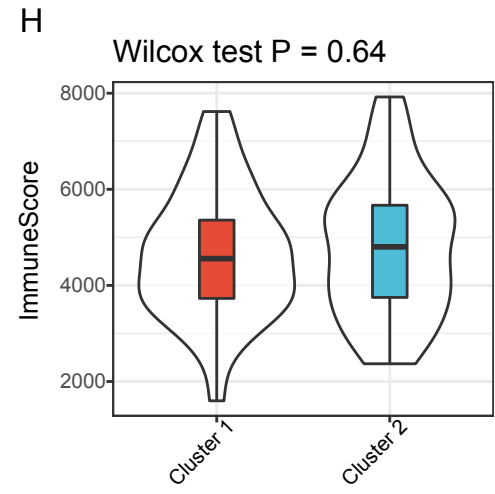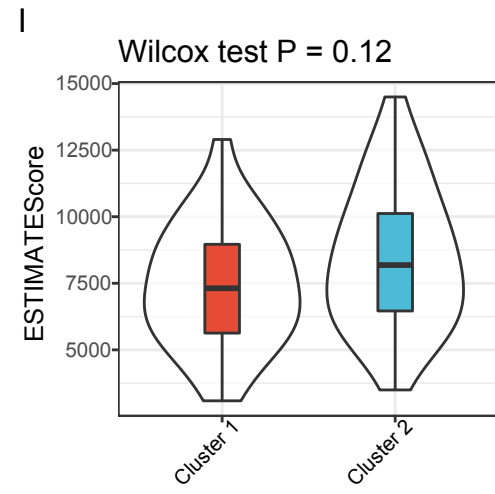

Group

Cluster 1 Cluster 2

Supplement: Supplementary file 2 — Additional file 2: Supplement Figure S2. The proportions of B cell, CD4+T cell, CD8+T cell, Neutrophil, Macrophage, Dendritic cell (DC), ImmuneScore, StromalScore, and ESTIMATEScore between the two subtypes. The enumeration of six tumor-infiltration immune cells was estimated using the “Tumor Immune Estimation Resource” (TIMER, https://cistrome.shinyapps.io/timer/) tool. [file 12885_2021_8030_MOESM2_ESM.pdf]

UBE2Z

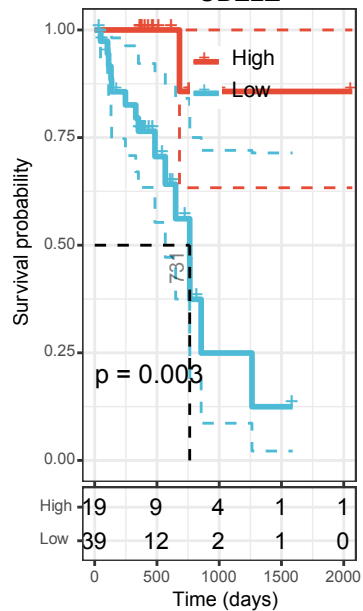

AMTN

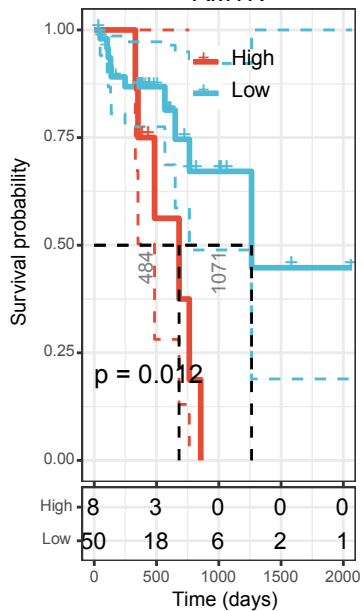

AK1

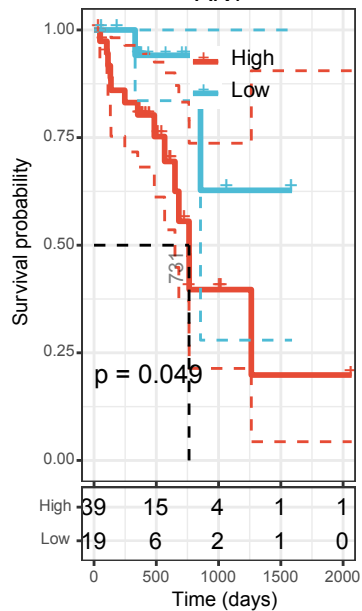

CDCA4

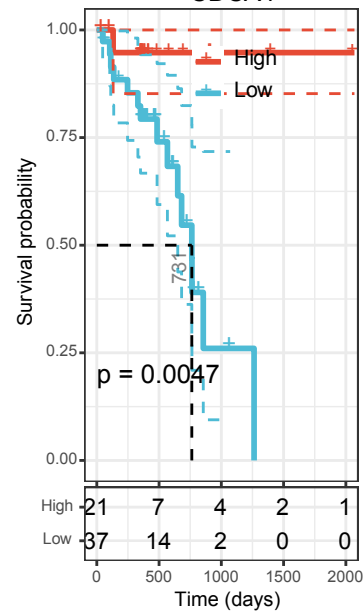

TLE1

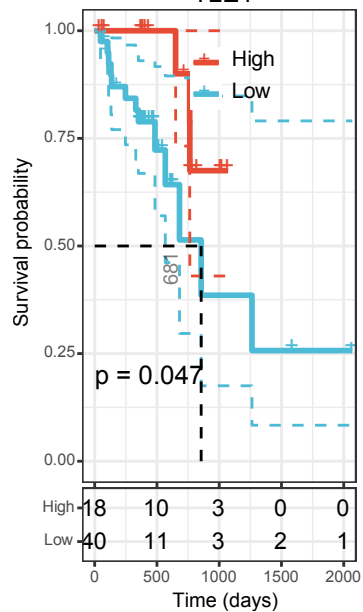

FXN

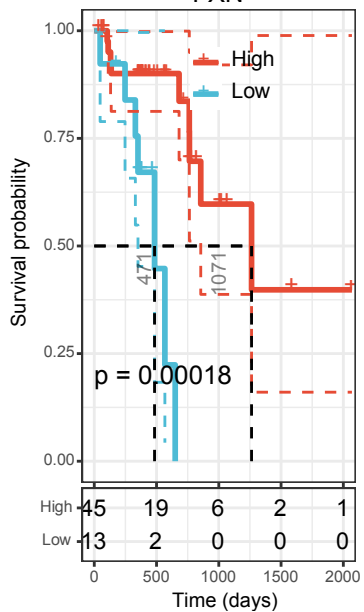

ZBTB6

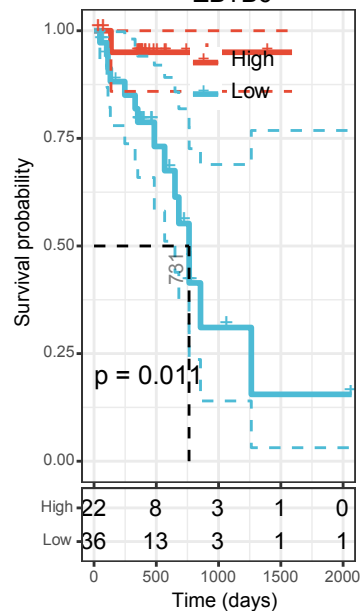

APLN

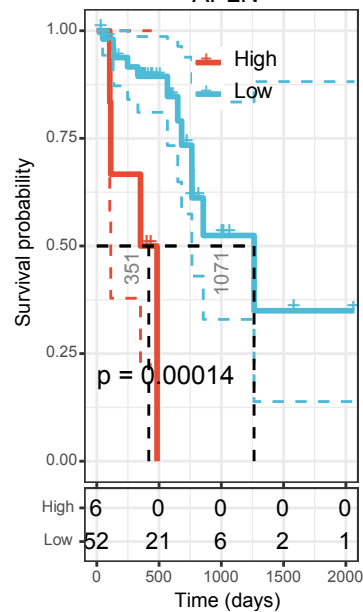

Supplement: Supplementary file 3 — Additional file 3: Supplement Figure S3. Kaplan-Meier analysis on the eight genes using the optimal cut-off value of the expression level of each gene. [file 12885_2021_8030_MOESM3_ESM.pdf]

A

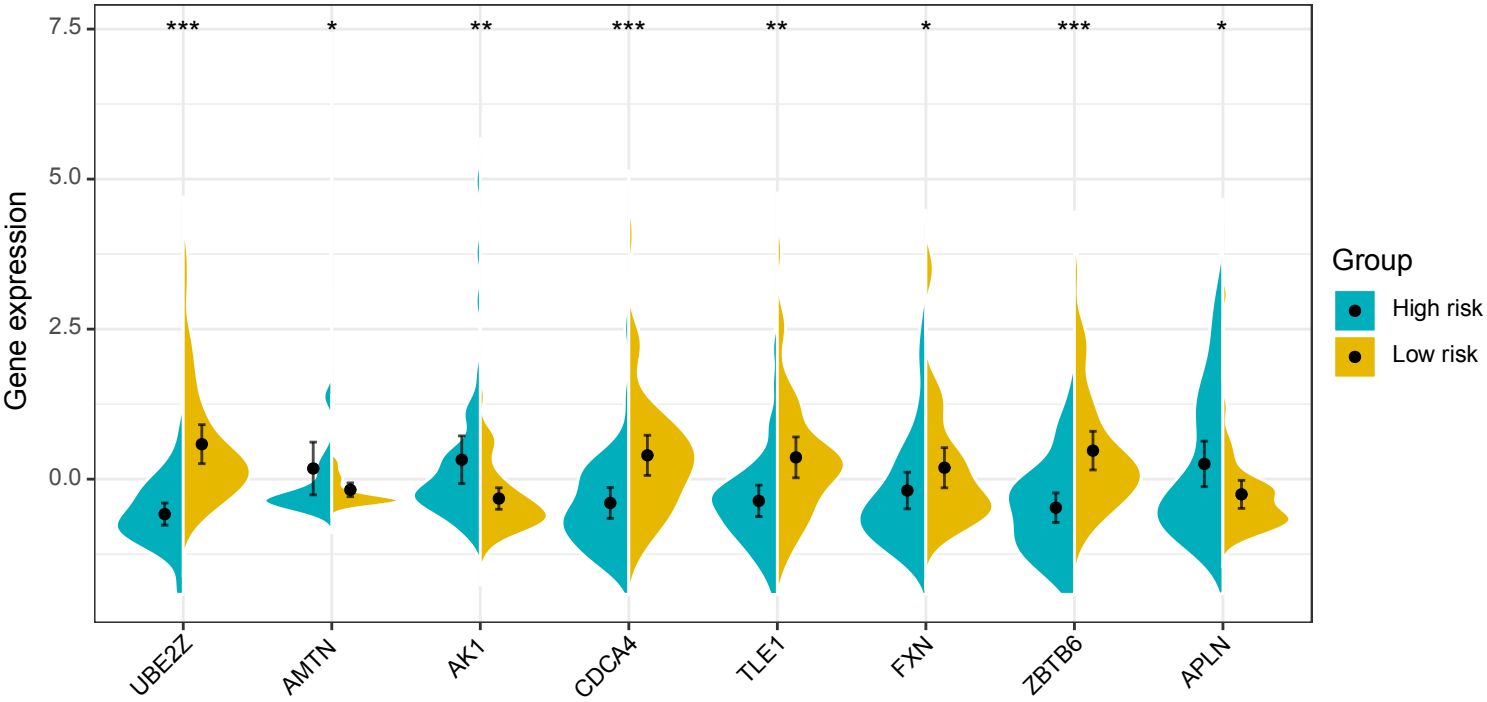

B

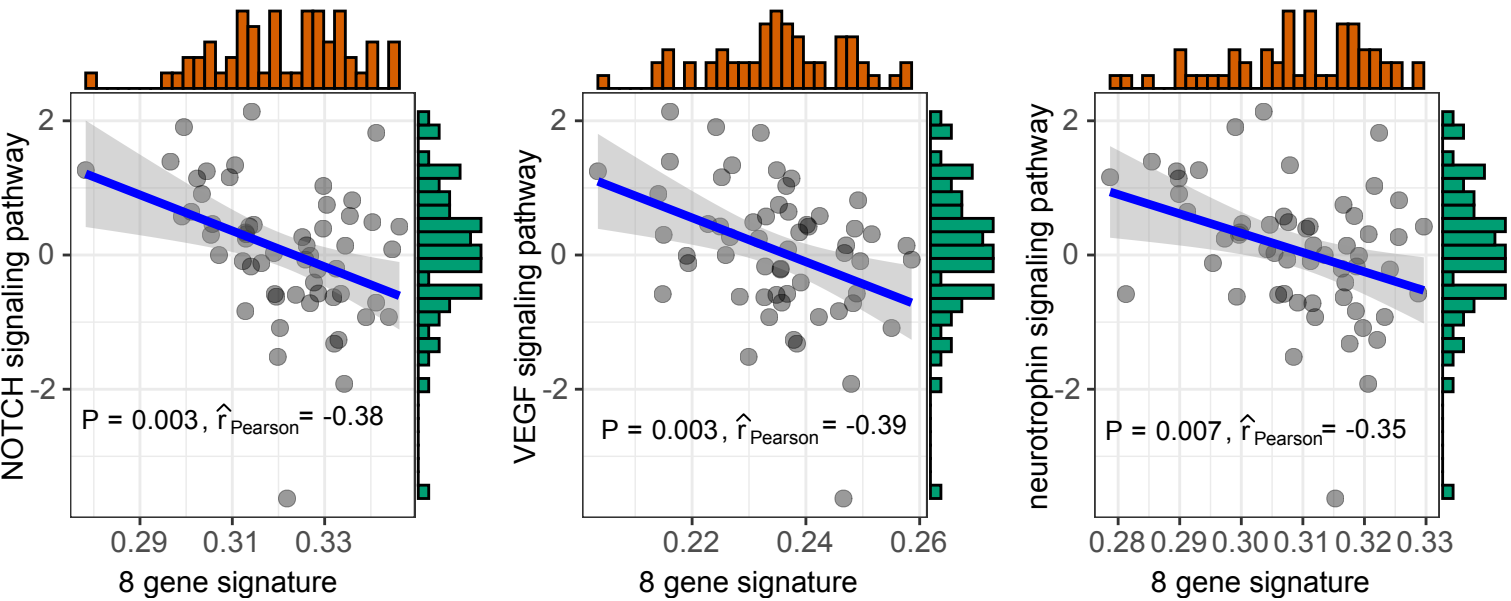

Supplement: Supplementary file 4 — Additional file 4: Supplement Figure S4. A. the differences on the expression level of these 8 gene between the two groups; B. single sample GSEA analysis results of pathways significantly associated with the 8-gene signature. [file 12885_2021_8030_MOESM4_ESM.pdf]

A

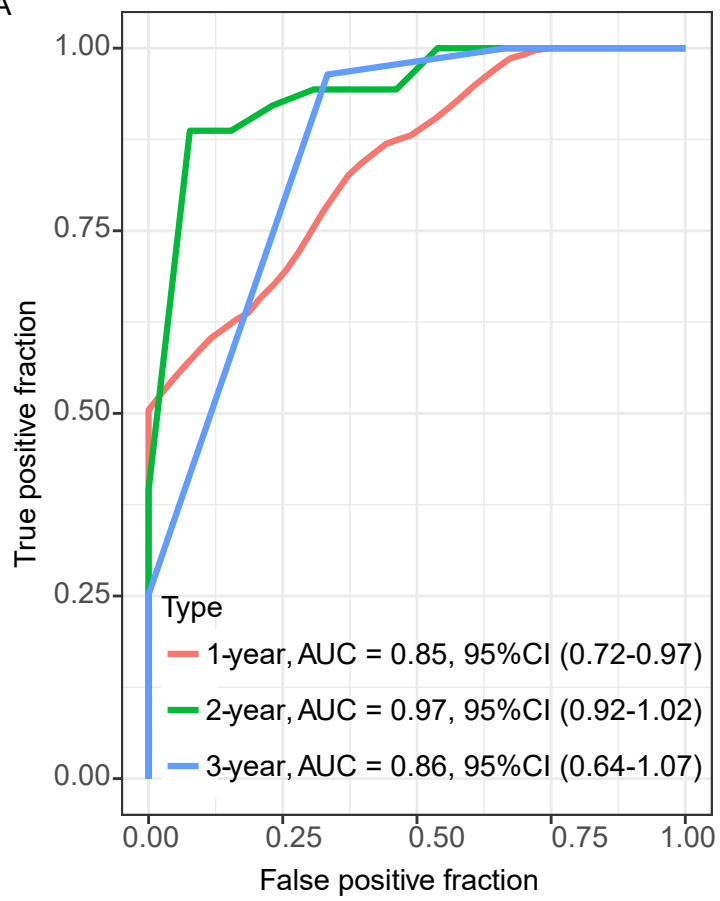

B

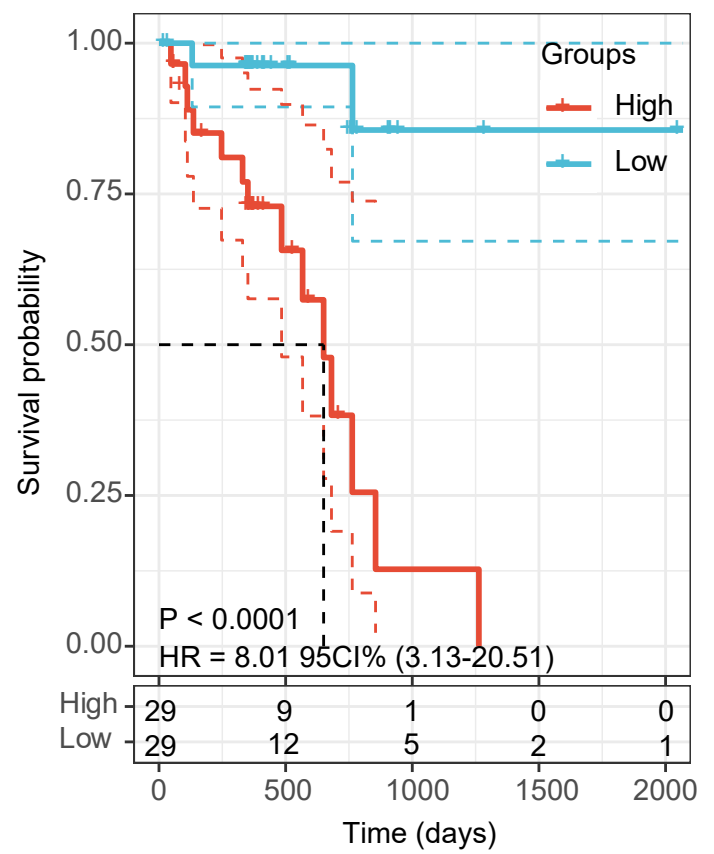

Supplement: Supplementary file 5 — Additional file 5: Supplement Figure S5. A. ROC curve of the 8-gene signature for 1-year, 3-year and 5-year DFS; B. Kaplan-Meier survival curve of DFS based on the 8-gene signature. ROC, receiver operating characteristic; AUC, area under the curve; HR, hazard ratio; CI, confidence interval. [file 12885_2021_8030_MOESM5_ESM.pdf]
